# A multicenter, retrospective chart review study comparing index therapy change rates in open-angle glaucoma or ocular hypertension patients newly treated with latanoprost or travoprost-Z monotherapy

**DOI:** 10.1186/1471-2415-11-13

**Published:** 2011-06-13

**Authors:** Joel M Fain, Sameer Kotak, Jack Mardekian, Jason Bacharach, Deepak P Edward, Steven Rauchman, Teresa Brevetti, Janet L Fox, Cherie Lovelace

**Affiliations:** 1 Pfizer Ophthalmics, Chicago, IL, USA; 2 Pfizer Ophthalmics, New York, NY, USA; 3 North Bay Eye Associates, Petaluma, CA, USA; 4 Summa Health System, Akron, OH, USA; 5 North Valley Eye, Mission Hills, CA, USA

## Abstract

**Background:**

Because latanoprost and the original formulation of travoprost that included benzalkonium chloride (BAK) have been shown to be similar with regard to tolerability, we compared initial topical intraocular pressure (IOP)-lowering medication change rates in patients newly treated with latanoprost or travoprost-Z monotherapy.

**Methods:**

At 14 clinical practice sites, medical records were abstracted for patients with a diagnosis of open-angle glaucoma or ocular hypertension and who were ≥40 years of age, had a baseline and at least one follow-up visit, and had no prior history of ocular prostaglandin use. Data regarding demographics, ocular/systemic medical histories, clinical variables, therapy initiations and reasons for changes, adverse events, and resource utilization were recorded from randomly chosen eligible charts. Primary outcomes were rates of and reasons for changing from the initial therapy within six months and across the full study period (1000 days).

**Results:**

Data from 900 medical charts (latanoprost, 632; travoprost-Z, 268) were included. For both cohorts, average follow-up was >1 year. Cohorts were similar with regard to age (median ~67 years), gender distribution (>50% female), and diagnosis (~80% with open-angle glaucoma). Within six months, rates of index therapy change for latanoprost versus travoprost-Z were 21.2% (134/632) and 28.7% (77/268), respectively (p = 0.0148); across the full study period, rates were 34.5% (218/632) and 45.2% (121/268), respectively (p = 0.0026). Among those who changed their index therapy, insufficient IOP control was the most commonly reported reason followed by adverse events; hyperemia was the most commonly reported adverse event at index therapy change.

**Conclusions:**

In this "real world" study of changes in therapy in patients prescribed initial monotherapy with latanoprost with BAK or travoprost-Z with SofZia, medication changes were common in both treatment groups but statistically significantly more frequent with travoprost-Z.

## Background

Research has demonstrated that progression of ocular hypertension to glaucoma and progression of glaucomatous damage can be delayed or halted with the use of topical ocular hypotensive agents [1-4]. Patients can benefit from these therapies only if they are taken as directed over the long term; however, medication discontinuation and changes may complicate patient management and make intraocular pressure (IOP) control problematic. Unfortunately, persistence with ocular hypotensives generally has been shown to be poor [5-11] although better with prostaglandin analogs than with agents in other classes [5,7,8,12]. Therapeutic interruptions may occur for many reasons. In patients treated with latanoprost, travoprost, or bimatoprost, the Glaucoma Adherence and Persistence Study (GAPS) [11] identified the need for additional IOP reduction and the presence of ocular adverse events, especially hyperemia, to be the main factors affecting continuation with therapy and medication changes.

All three prostaglandins evaluated in the GAPS [11] were preserved with benzalkonium chloride (BAK). Currently the most widely used preservative in ocular hypotensive formulations, BAK has been in use for more than 50 years [13,14]. While it has been suggested that preservative-free formulations could improve ocular tolerability and thereby reduce treatment discontinuation [15], such formulations pose their own risks because preservatives are added to multiple-use containers of ophthalmic preparations in order to prevent bacterial contamination [16] and to reduce the risk of serious infections such as infectious keratitis [14]. Moreover, the contribution of BAK to ocular toxicity remains unclear. While animal studies [17-20] and studies of cultured corneal [21] and conjunctival cells [22] have reported dose-dependent, BAK-induced epithelial cellular damage, these findings may not accurately reflect ocular surface conditions in humans, and the levels of BAK contained in ophthalmic solutions are not likely to cause clinically significant adverse corneal effects [23-27].

An alternative preservative, SofZia^®^, currently is used as the preservative in travoprost-Z. Although latanoprost with BAK has been found to exhibit more effective microbial protection than travoprost-Z [28], the question of whether SofZia improves tolerability has not been resolved. For example, a randomized, double-masked comparison of travoprost with BAK versus travoprost-Z found the formulations were equivalent with regard to tolerability and the occurrence of ocular adverse events [29], and a comparison of the ocular surface tolerability of latanoprost and bimatoprost preserved with BAK versus travoprost-Z found no significant differences in objective clinical measures of ocular tolerability [30]. In contrast, results of an in vitro study of corneal epithelial cells suggested that SofZia may be less toxic than BAK [31].

Because latanoprost and the original formulation of travoprost that included BAK have been shown to be similar with regard to tolerability [32,33], we compared rates of therapy change in patients with open-angle glaucoma or ocular hypertension who were newly treated with latanoprost or travoprost-Z as monotherapy.

## Methods

This retrospective multicenter, medical chart review was conducted at 14 geographically diverse sites in the United States. Prior to including a site, a Pfizer Regional Medical & Research Specialist conducted an on-site investigator meeting to review the study protocol and chart review instructions with the investigator and the research coordinators/technicians responsible for chart selection and data abstraction. Training was provided, and coordinators/technicians abstracted a standardized dummy chart to ensure consistent and accurate data collection and documentation. Additional training was provided as needed to ensure consistency between sites. Because this was a retrospective study, it did not require approval from an ethics committee. Each research site de-identified data on case report forms using numeric codes to assure patient confidentiality.

Records for patients initiating either latanoprost or travoprost-Z monotherapy for the treatment of bilateral primary open-angle glaucoma or ocular hypertension between October 1, 2006, and September 8, 2009, were reviewed. Eligible patients were ≥40 years of age, had no prior history of ocular prostaglandin use and had charted data reflecting at least one follow-up visit during the six months following the baseline (treatment initiation) visit. Exclusion criteria included prescription of any ocular hypotensive medication in the six months prior to the baseline visit; concomitant diagnosis of closed-angle glaucoma; participation in an ophthalmology-related clinical trial in the three months prior to baseline; intraocular surgery prior to baseline; and the existence of any clinical condition that would contribute to discontinuation (e.g. ocular infection, allergy).

Each site was asked to randomly choose an alphabetical starting point and, beginning with October 1, 2006, to identify consecutive charts that met all inclusion and no exclusion criteria. Sites were to review consecutive charts until records for 60 patients who initiated latanoprost monotherapy and 30 who initiated travoprost-Z monotherapy were identified. The unbalanced sample size reflected the fact that travoprost-Z was introduced only in 2006 and was prescribed less frequently than latanoprost which has been available since 1996. Once the target number for one therapy cohort was reached, the site was to continue to select consecutive eligible charts for the remaining cohort only. The smallest site contributed 20 patients and the largest site contributed 92 patients.

Demographic data (age, gender) were recorded. For baseline and all follow-up visits, the visit date; diagnosis; IOP level; visual field defect; cup-to-disc ratio; central corneal thickness; and ocular comorbidities, diagnostic tests, and medications were documented. All reported adverse events were recorded. An adverse event was classified as "serious" if it was life-threatening, required inpatient hospitalization/prolongation of hospitalization, resulted in persistent or significant disability/incapacity, or caused congenital anomaly/birth defect or death.

Data were collected from baseline until the end of the observation period (i.e. last visit) in the charts. The time to index therapy change was analyzed at month 6 (180 days) and at the end of the observation period (i.e. last visit in chart). Research coordinators/technicians responsible for chart selection and data abstraction were specifically instructed to exclude patients with disruption in therapy due to forced formulary changes (switches) during the follow-up period. Patients with changes due to other access or cost issues, such as those with prescription coverage who requested less expensive generic products, were included; for example, higher copays for branded versus generic drugs.

The statistical significance of between-cohort differences in categorical variables was tested using the chi-square test and in continuous variables using the two-sample t-test. All tests were two-tailed with a significance level of p < 0.05.

Change in the index therapy could reflect any of the following: (1) add-on: prescription for an ocular hypotensive agent in addition to the initial monotherapy; (2) switch: switch from the index monotherapy to another ocular hypotensive agent; (3) discontinuation: discontinuation of ocular hypotensive therapy; and (4) surgery/procedure: documentation of a glaucoma-related surgery or procedure. Unadjusted Cox proportional hazards models were used to estimate the hazard of index therapy change between treatment cohorts from baseline to month six and from baseline to the end of the study. Kaplan-Meier survival curves graphically represented the probability of changing the index therapy throughout the follow-up periods.

Based on results of the GAPS [11] and assuming that travoprost and travoprost-Z have similar tolerability profiles, a sample of 659 charts of latanoprost-treated patients and 329 charts of those treated with travoprost-Z was estimated to provide an 80% power to demonstrate a meaningful difference between latanoprost (4.2% change rate) and travoprost-Z (9.0% change rate) at a 0.05 significance level.

## Results

In all, charts for 632 patients initiating monotherapy with latanoprost and for 268 treated initially with travoprost-Z monotherapy were abstracted. Baseline demographic characteristics and medical histories are summarized in Table 1. In both cohorts, the median age was approximately 67 years, >50% were female, and 80% were diagnosed with primary open-angle glaucoma. Systemic comorbidities at baseline were common with more than half of the patients in each cohort reporting hypertension and approximately one-quarter reporting a lipid disorder. At baseline, nearly 30% in each cohort had undergone cataract surgery, and approximately 10% reported a history of dry eye. Baseline IOP values were similar across treatment groups in both eyes.

**Table 1:** Baseline demographic characteristics and medical history*

	Latanoprost N = 632	Travoprost-Z N = 268
**Age, years**		
Mean ± SD	66.7 ± 12.8	66.9 ± 11.1
Median (range)	67.0 (32, 97)	66.5 (42, 96)
**Gender**		
Male	284 (45.0)	126 (47.6)
Female	347 (55.0)	139 (52.5)
**Diagnosis**		
Primary open-angle glaucoma	499 (80.0)	214 (79.9)
Ocular hypertension	119 (19.3)	52 (19.5)
**Family history of glaucoma**		
Yes	131 (20.7)	65 (24.3)
No	501 (79.3)	203 (75.8)
**Systemic comorbidities**^**†**^		
Hypertension^‡^	322 (51.0)	159 (59.3)
Lipid disorder	170 (26.9)	60 (22.4)
Diabetes^‡^	108 (17.1)	65 (24.3)
Thyroid disease	76 (12.0)	24 (9.0)
Allergic rhinitis	50 (7.9)	17 (6.3)
Asthma	40 (6.3)	19 (7.1)
Depression	39 (6.2)	12 (4.5)
Other	310 (49.1)	121 (45.2)
**Coexisting ocular conditions**^**†**^		
Cataract surgery	173 (27.4)	80 (29.9)
Dry eye	83 (13.1)	27 (10.1)
Macular degeneration	42 (6.7)	13 (4.9)
Diabetic retinopathy	17 (2.7)	9 (3.4)
Seasonal allergic conjunctivitis	7 (1.1)	6 (2.2)
Other	319 (50.5)	154 (57.5)
Intraocular pressure^§^		
Right eye		
Mean ± SD	22.9 ± 6.2	23.1 ± 6.7
Median	22	22
Left eye		
Mean ± SD	22.4 ± 5.4	22.5 ± 5.5
Median	22	22

SD = standard deviation.

*N (%) unless otherwise indicated.

^†^Reported by ≥2% of patients in either cohort.

^‡^p < 0.05.

^§^Latanoprost, N = 629; travoprost-X, N = 267.

Ocular procedures and ocular surface tests were performed with similar frequency across cohorts at the baseline visit (Table 2). As would be expected, records for virtually all patients included a notation of IOP level, while results of perimetry, ophthalmoscopy, gonioscopy, and measurement of central corneal thickness were recorded for approximately half of the patients overall. At baseline, ocular surface tests were performed relatively infrequently in both cohorts. Over the full study period, the mean ± standard deviation number of office visits was 3.9 ± 2.3 for latanoprost and 4.1 ± 2.7 for travoprost-Z (p = 0.20), and the duration of follow-up was similar between cohorts (latanoprost: 405.7 ± 257.5 days, range 5 to 1034 days; travoprost-Z: 397.8 ± 248.2 days, range 8 to 1000 days).

**Table 2:** Ocular procedures and ocular surface tests performed at baseline, N (%)*

	Latanoprost N = 632	Travoprost-Z N = 268
**Ocular procedures**		
IOP	627 (99.2)	265 (98.9)
Perimetry	344 (54.4)	132 (49.3)
Ophthalmoscopy	330 (52.2)	143 (53.4)
Gonioscopy	303 (47.9)	147 (54.9)
Central corneal thickness measured^†^	280 (44.3)	144 (53.7)
Photography^†^	130 (20.6)	33 (12.3)
Heidelberg retinal tomography	154 (24.4)	55 (20.5)
Optical coherence tomography	97 (15.4)	55 (20.5)
GDx nerve fiber analysis	31 (4.9)	16 (6.0)
**Ocular surface tests**		
Tear breakup time	57 (9.0)	28 (10.5)
Staining	13 (2.1)	4 (1.5)

*Reported by ≥2% of patients in either cohort.

^†^p < 0.05.

**Figure 1:**
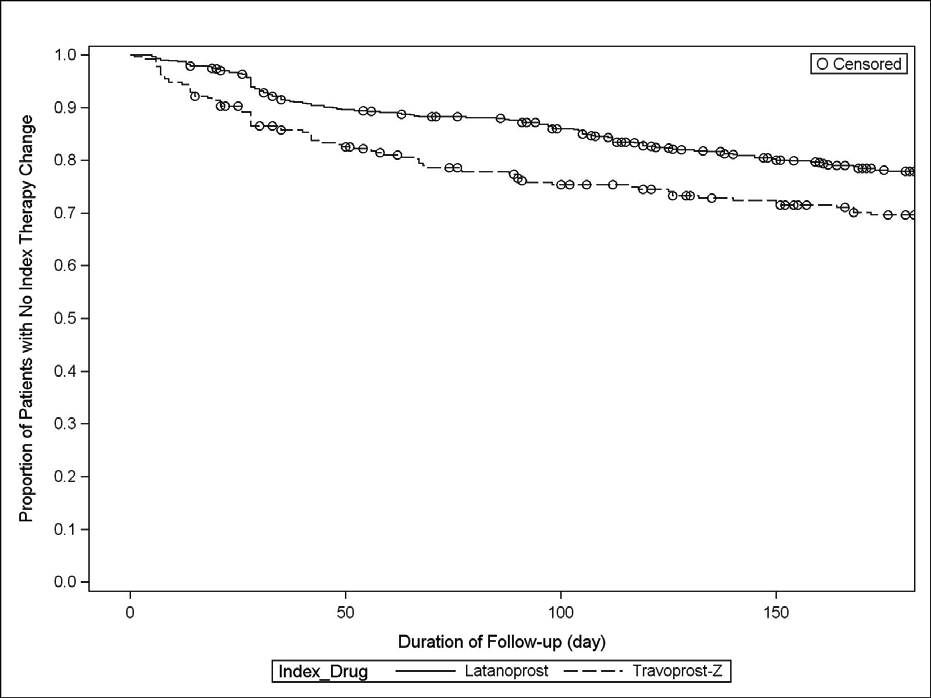
**Kaplan-Meier curves of days to index therapy change within 6 months**.

**Figure 2:**
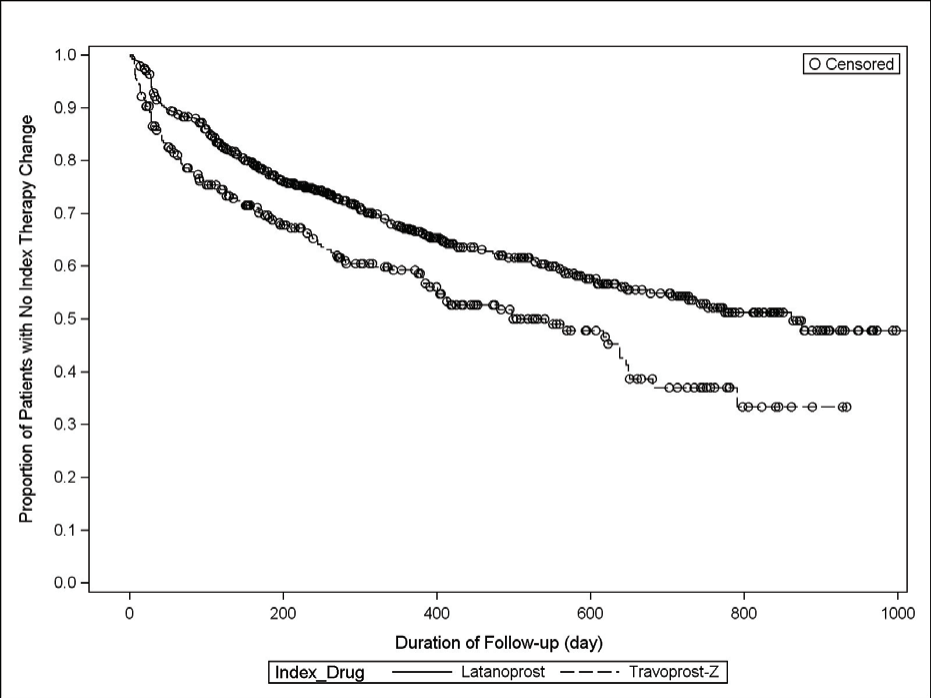
**Kaplan-Meier curves of days to index therapy change across the full study period**.

Within six months, rates of index therapy change for latanoprost versus travoprost-Z were 21.2% (134/632) and 28.7% (77/268), respectively (p = 0.0148); across the full study period, rates were 34.5% (218/632) and 45.2% (121/268), respectively (p = 0.0026; Table 3). Patients initially treated with travoprost-Z were 52% more likely (hazard ratio [HR]: 1.52; 95% confidence interval [CI]:1.15-2.00; p = 0.0035) to have an index therapy change within the first six months and 50% more likely (HR: 1.50; 95% CI: 1.20-1.87; p = 0.0004) to experience an index therapy change during the study compared with patients treated first with latanoprost. Among those changing therapy, days to therapy change was longer among those initially treated with latanoprost. The time in days to index therapy change are shown in Kaplan-Meier curves for the two ocular hypotensive therapies in Figures 1 and 2.

**Table 3:** Index therapy change within 6 months and full study period

	Within 6 months		Across full study period	
	
	Latanoprost N = 632	Travoprost-Z N = 268	Latanoprost N = 632	Travoprost-Z N = 268
Changed therapy, n %	134 (21.2)	77 (28.7)	218 (34.5)	121 (45.2)
p-value*	0.0148		0.0026	
Hazard ratio^†^	1.519		1.499	
p-value	0.0035		0.0004	
Days to change				
Mean ± SD	72.9 ± 51.2	54.1 ± 48.0	194.8 ± 192.9	178.0 ± 196.7
Median	56.5	40.0	125.0	90.0

SD = standard deviation.

*Chi-square test.

^†^The reference cohort is latanoprost. Based on the Cox proportional hazards model.

Among those who changed their index therapy, switch to a different ocular hypotensive agent was the most frequent type of change in both time periods (>40% of changes), followed by the addition of ocular hypotensive therapy (>26% of changes; Table 4). The most frequently cited reason for change was insufficient IOP control reported for >60% of those changing therapy within the first six months and for >50% of those changing across the full study period (Table 4; patients with therapy disruption related to access or formulary changes were excluded from chart abstraction). The second most frequently cited reason was the occurrence of adverse events. Hyperemia was the adverse advent charted most frequently at the time of the first index therapy change in both measurement time periods and was reported approximately four times more frequently among those treated with travoprost-Z (p < 0.0001 for both within six months and across the full study period; Table 5). Dry eye was reported infrequently, in 1/134 patients in the latanoprost group and 1/77 patients in the travoprost-Z group, and was not reported as a reason for withdrawal by any patient. When those who switched therapy due to insufficient IOP control were excluded, time to index therapy change was longer among those treated initially with latanoprost during both the first six months and the full study period (p = 0.0523 and p = 0.0427, respectively).

**Table 4:** Type of and reason for index therapy change within 6 months and full study period among patients who changed, N (%)

	Within 6 months		Across full study period	
	
	Latanoprost N = 134	Travoprost-Z N = 77	Latanoprost N = 218	Travoprost-Z N = 121
**Type of change**				
Add-on	36 (26.9)	26 (33.8)	62 (28.4)	40 (33.1)
Switch	65 (48.5)	36 (46.8)	102 (46.8)	50 (41.3)
Discontinuation	14 (10.5)	5 (6.5)	23 (10.6)	13 (10.7)
Surgery/procedure	19 (14.2)	10 (13.0)	31 (14.2)	18 (14.9)
Reason for change*				
IOP not controlled	83 (61.9)	50 (64.9)	119 (54.6)	76 (62.8)
Adverse events	16 (11.9)	16 (20.8)	24 (11.0)	18 (14.9)
Physician preference	11 (8.2)	4 (5.2)	21 (9.6)	9 (7.4)
Non-compliance	8 (6.0)	2 (2.6)	11 (5.0)	6 (5.0)
Cost	9 (6.7)	3 (3.9)	19 (8.7)	4 (3.3)
Patient request	5 (3.7)	2 (2.6)	10 (4.6)	4 (3.3)
Ocular nerve head				
changes	1 (0.7)	0	6 (2.8)	1 (0.8)
Other	11 (8.2)	3 (3.9)	25 (11.5)	5 (4.1)

IOP = intraocular pressure.

*Reported by ≥2% of patients in either cohort at either time point. More than one reason could be reported for each patient.

**Table 5:** Adverse event(s) charted at index therapy change within 6 months and full study period among patients who changed, N (%)

	Within 6 months		Across full study period	
	
	Latanoprost N = 134	Travoprost-Z N = 77	Latanoprost N = 218	Travoprost-Z N = 121
Hyperemia^†^	7 (5.2)	18 (23.4)	10 (4.6)	21 (17.4)
Burning	7 (5.2)	2 (2.6)	10 (4.6)	2 (1.7)
Pain/ocular discomfort	4 (3.0)	3 (3.9)	4 (1.8)	4 (3.3)
Blepharitis	3 (2.2)	2 (2.6)	4 (1.8)	2 (1.7)
Dry eye	1 (0.7)	1 (1.3)	3 (1.4)	4 (3.3)
Foreign body sensation	1 (0.7)	2 (2.6)	2 (0.9)	4 (3.3)
Pruritis	1 (0.7)	0	7 (3.2)	1 (0.8)
Other	8 (6.0)	8 (10.4)	16 (7.3)	10 (8.3)

*Reported by ≥2% of patients in either cohort at either time point. More than one event could be reported for each patient.

^†^p < 0.0001 for both within six months and across full study period.

## Discussion

Although we hypothesized that the rate of index therapy change would be lower with travoprost-Z preserved with SofZia than with latanoprost preserved with BAK, we found change rates to be statistically significantly lower in latanoprost-treated patients both within six months and across the full study period. As in studies of persistency with ocular hypotensive agents generally, rates of uninterrupted use were lower than desirable for both latanoprost and travoprost-Z, but patients initially prescribed travoprost-Z were approximately 50% more likely to change therapy both within the first 6 months and across the full study period (p < 0.01 for both comparisons with latanoprost). Combining proportions of patients who either switched or discontinued to estimate how many patients completely stopped taking the initial therapy revealed that, within the first six months, 12.5% (79/632) of latanoprost-treated patients and 15.3% (41/268) of those treated with travoprost-Z stopped the initial therapy and that 19.8% (125/632) and 23.5% (63/268), respectively, did so across the full study period. Similar proportions of patients in the two treatment groups changed due to a glaucoma-related surgery or procedure (approximately 14% at each measurement time point).

Our finding of greater persistence with latanoprost monotherapy than with travoprost-Z monotherapy contrasts with results of retrospective claims database analyses of prostaglandin analog treatment patterns [34-36]. In those studies, rates of adjunctive IOP-lowering therapy use favored travoprost-Z, with differences between latanoprost and travoprost-Z ranging from 7.6% over 12 months (16.5% vs 8.9%, respectively) [34] to 2% over two years (37% vs 35%, respectively) [35]. Directly comparing findings of the present study and the published claims database analyses is difficult given differences in research questions, definitions of outcomes, and statistical analyses. In particular, the studies by Schmier et al [34-36] included only newly initiating patients who remained on the index prostaglandin for least 12 or 24 months (depending on the study), while the present research identified patients newly prescribed an ocular prostaglandin and tracked therapy changes throughout the follow-up period. Nevertheless, such contrasting results suggest that additional research is needed to evaluate whether the low levels of BAK contained in ophthalmic solutions may cause significant adverse corneal effects that impact patient persistence.

Changes in ocular hypotensive therapy may be precipitated by a number of factors. In the GAPS [11] with an average duration of chart review of 4.1 years, lack of efficacy was the most common reason cited by physicians for switching medication followed by adverse events (43% vs 19%, respectively). Among patients who changed therapy in the present study, uncontrolled IOP was noted as a reason for change within the first six months in >60% charts and as a reason for nearly 55% of changes from latanoprost and 63% of changes from travoprost-Z across the full study period. As in the GAPS [11], adverse events were the second most frequently cited reason for change, noted in between 11% and 21% of cases; changes were attributed to adverse events in somewhat greater proportions of those treated with travoprost-Z.

The most frequently recorded adverse event was hyperemia, which was noted significantly more frequently with travoprost-Z than with latanoprost at both measurement time points. In the GAPS [11], hyperemia also was the most common adverse event. In that study, of the 195 patients with charted adverse events, hyperemia was noted in 135 (69%) records, and an episode of hyperemia occurred in a significantly greater proportion of those exposed to travoprost (35%) than to latanoprost (22%; p = 0.0123). It has been suggested that BAK exposure is related to an increased incidence of dry eye [15], but therapy withdrawals from dry eye were not obvious in the latanoprost with BAK group.. This lack of association between these agents and the incidence of dry eye parallel the results of a retrospective analysis of three large prescription databases that found no significant difference in the incidence of ocular surface disease (defined as dry eye or ocular infection) between patients prescribed latanoprost versus travoprost-Z (14.0% vs 14.4%, respectively; p = 0.45) [37].

The American Academy of Ophthalmology [38,39] and the European Glaucoma Society [40] have produced guidelines for the evaluation and treatment of patients with glaucoma or ocular hypertension, but eye care professionals may not routinely follow these recommendations [41-44]. In the GAPS [44], IOP and results of disc evaluations and imaging and of visual field tests were recorded in charts of 90% of open-angle glaucoma patients; in contrast, chart notations of central corneal thickness measurement, gonioscopy, and establishment of an IOP target level were present for about half of patients. In the present study, we tabulated frequencies of ocular procedures and ocular surface tests performed at baseline. An IOP measurement was recorded for nearly all (>98%) patients in both treatment groups and about half of charts included findings of perimetry, ophthalmoscopy, gonioscopy, and central corneal thickness measurement. Other procedures and ocular surface tests were performed less frequently.

A major strength of the current study was the large number of charts reviewed (N = 900). However, the follow-up time frame was too short to support assessments of changes in parameters such as visual field. Rates of therapy "gaps" and restarts, which have been documented using large medical/pharmacy databases [10,11], cannot be reliably inferred from medical records. Although the 14 sites from which data were abstracted were geographically diverse, they may not represent the full population of ophthalmology practices. Every effort was made to ensure the consistency of chart abstraction across sites, but no formal tests of reliability were undertaken. In addition, time to and reasons for changing medication were not cross-validated by an independent committee and were not evaluated in a masked fashion. Insufficient IOP reduction was the most commonly reported reason for change, but IOP changes at the patient level were not assessed and could be considered in future analyses. Finally, because latanoprost and the original formulation of travoprost with BAK have similar tolerability profiles [32,33], we hypothesized that any improvement in the tolerability of travoprost with SofZia would lead to lower rates of therapy change in comparison to latanoprost with BAK. Although we did not find lower rates of therapy change among those treated with travoprost-Z, future research might expand the study by comparing persistence with BAK-preserved travoprost, bimatoprost, and latanoprost versus ocular hypotensive formulations without BAK - including travoprost with SofZia

## Conclusion

In this "real world" study of changes in therapy in patients prescribed initial monotherapy with latanoprost with BAK or travoprost-Z with SofZia, medication changes were common in both treatment groups but statistically significantly more frequent with travoprost-Z.

## Competing interests

JMF, SK, JM, and JLF are employees of Pfizer Inc, New York, NY. TB and CL were employees of Pfizer Inc, New York, NY at the time the research was conducted, JB, DPE, and SR were paid consultants to Pfizer in connection with the development of this manuscript. The study was sponsored by Pfizer Inc, New York, NY.

## Authors' contributions

JMF, SK, JM, TB, JLF, and CL participated in development of the study concept and design. JB, DPE, and SR participated in acquisition of data. JMF, SK, and JM participated in the analysis and interpretation of data and drafting of the manuscript. Study supervision was provided by JMF, SK, TB, JLF, and CL. All authors critically reviewed the manuscript for important intellectual content, and all authors read and approved the final manuscript.

## Pre-publication history

The pre-publication history for this paper can be accessed here:

http://www.biomedcentral.com/1471-2415/11/13/prepub
